# Resolving Hot Spots in the C-Terminal Dimerization Domain that Determine the Stability of the Molecular Chaperone Hsp90

**DOI:** 10.1371/journal.pone.0096031

**Published:** 2014-04-23

**Authors:** Emanuele Ciglia, Janina Vergin, Sven Reimann, Sander H. J. Smits, Lutz Schmitt, Georg Groth, Holger Gohlke

**Affiliations:** 1 Institute for Pharmaceutical and Medicinal Chemistry, Heinrich-Heine-University, Düsseldorf, Germany; 2 Institute for Biochemical Plant Physiology, Heinrich-Heine-University, Düsseldorf, Germany; 3 Institute of Biochemistry, Heinrich-Heine-University, Düsseldorf, Germany; Fred Hutchinson Cancer Research Center, United States of America

## Abstract

Human heat shock protein of 90 kDa (hHsp90) is a homodimer that has an essential role in facilitating malignant transformation at the molecular level. Inhibiting hHsp90 function is a validated approach for treating different types of tumors. Inhibiting the dimerization of hHsp90 via its C-terminal domain (CTD) should provide a novel way to therapeutically interfere with hHsp90 function. Here, we predicted hot spot residues that cluster in the CTD dimerization interface by a structural decomposition of the effective energy of binding computed by the MM-GBSA approach and confirmed these predictions using *in silico* alanine scanning with DrugScore^PPI^. Mutation of these residues to alanine caused a significant decrease in the melting temperature according to differential scanning fluorimetry experiments, indicating a reduced stability of the mutant hHsp90 complexes. Size exclusion chromatography and multi-angle light scattering studies demonstrate that the reduced stability of the mutant hHsp90 correlates with a lower complex stoichiometry due to the disruption of the dimerization interface. These results suggest that the identified hot spot residues can be used as a pharmacophoric template for identifying and designing small-molecule inhibitors of hHsp90 dimerization.

## Introduction

Protein-protein complexes have gained increasing attention in structural biology and drug discovery due to their ubiquitous participation in fundamental cellular processes. As such, protein-protein interactions (PPIs) are involved in a variety of physiological regulatory mechanisms, e.g., signaling, cellular growth, and apoptosis [1], [2]. PPIs also play an important role in pathophysiology [3], [4] such that modulating PPIs is considered a valuable approach for treating diseases [2], [3], [5]–[7]. Targeting PPIs is considered difficult, however, because of the size, lack of deep binding pockets, and stability of PPIs. Yet, protein-protein interfaces have been shown to be energetically non-homogeneous in that only a few “hot spot” residues account for most of the binding affinity [8]–[10]. Accordingly, PPI modulators often target only the functional epitope that contains these hot spots [11]–[13]. Thus, identifying such hot spots provides important insights into the energetics of PPIs, which can be exploited for the identification of PPI modulators [12].

Here, we aim at resolving hot spots in the C-terminal dimerization domain of the human heat shock protein of 90 kDa (hHsp90). Hsp90 is a molecular chaperone that belongs to a highly conserved family of proteins that are central to a number of cellular functions, including protein (re)folding, stabilization, and quality control [14]–[16]. Despite its high basal expression in eukaryotes and prokaryotes [17], [18], Hsp90 remains mostly in a latent state under physiological conditions. In response to environmental stress, the cellular activity of Hsp90 (along with other heat shock proteins) is increased in order to protect the exposed cell [16], [19]. Recent data has also demonstrated essential roles for chaperones in facilitating malignant transformation at the molecular level: the chaperone allows tumor cells to tolerate mutations in multiple critical signaling molecules that would otherwise be lethal [20], [21]. Accordingly, many studies have validated Hsp90 inhibition as an approach for treating different types of tumors [14], [22]–[26].

**Figure 1:**
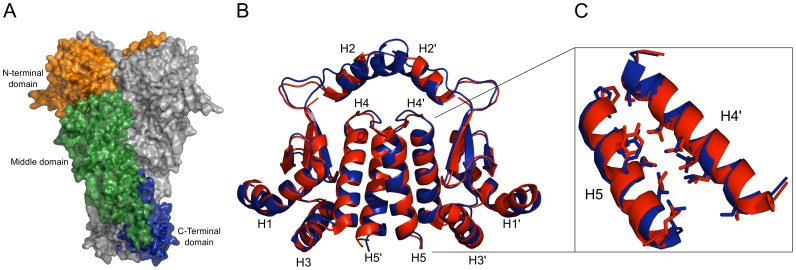
Homology model. (A) Surface representation of the full length *S. cerevisiae* Hsp90 (PDB code 2CG9), showing the three different protein domains (N-terminal domain: orange, middle domain: green, C-terminal domain: blue). (B) Homology model of hHsp90 C-terminal domain (blue) overlaid with a crystal structure (PDB code 3Q6M) of the same domain (red) (C) Blow-up of the overlay highlighting the side chain orientation of residues located at the interface of helices H5 and H4′.

Regarding its structure, Hsp90 is a flexible homodimeric protein; each monomer consists of three major domains: an amino terminal domain (NTD), a middle domain (M), and a carboxy terminal domain (CTD) [17], [27] (Figure 1, A). The NTD contains a nucleotide binding pocket, responsible for Hsp90's ATPase activity, which is coupled to the chaperone activity [28], [29]. This pocket is the binding site of most of the known Hsp90 inhibitors [30], [31]. The M domain is the major interaction site for Hsp90 clients, and bridges NTD and CTD [28]. In addition to being involved in regulating ATPase activity and co-chaperone recruitment, the CTD is responsible for Hsp90 dimerization [18], [32]. The dimerization interface is formed by two pairs of helices creating a characteristic four helix bundle [17], [33]. Recent results showed that the C-terminal dimer opens and closes with fast kinetics [34] in contrast to previous assumptions that the C-terminal interface is permanently dimerized [17]. These findings led us to hypothesize that inhibiting the C-terminal dimerization will be a viable way to interfere with Hsp90 activity. Although some Hsp90 inhibitors have been described that act on the CTD [35], [36] to the best of our knowledge none of these targets the dimerization interface.

In order to identify hot spots as a first step to define the functional epitope in the dimerization interface, we conducted a combined computational and experimental study. First, we predicted potential hot spot candidates by two independent computational approaches, MM-GB/SA [37] and DrugScore^PPI^ [38], [39], using a homology model of the human C-terminal Hsp90 domain. A subset of these was mutated to alanine, and the stability of wild type and mutant proteins was evaluated by a Thermofluor assay [40], size exclusion chromatography (SEC), and multi-angle light scattering (MALS). Our findings provide insights into the energetics of CTD dimerization in Hsp90, which are valuable for pursuing a novel approach that aims at therapeutically interfering with Hsp90 activity.

## Results

### Homology modeling and molecular dynamics simulations

When starting this study, neither a crystal structure of the human full length Hsp90 (hHsp90) nor of its CTD was available, which would be required for any later structure-based endeavor to identify PPI modulators. Thus, we set out to generate a model of the hHsp90 CTD by comparative modeling with MODELLER [41] using crystal structures from *S. cerevisiae* and *E. coli* as templates. The sequence identity (similarity) between the target sequence and the template sequences is sufficiently high (*S. cerevisiae*: 54% (74%); *E. coli*: 25% (43%); (Figure S1 in File S1). The obtained model is of good structural quality as assessed with the PROCHECK software [42] (Figure S2 in File S1). Recently, a crystal structure of an M-CTD construct of hHsp90 has been reported (PDB code: 3Q6M) [43]. Our model and the crystal structure show very good structural agreement as demonstrated by a root mean square deviation (RMSD) of all C_α_ atoms of ∼0.8 Å (Figure 1, B). This value decreases to ∼0.7 Å when the C_α_ atoms of only the amino acids located in the four helix bundle (helices H4, H4′ and H5, H5′) are taken into account (Figure 1, C). The orientation of the side chains in the dimerization interface agrees almost perfectly between the model and the crystal structure (Figure 1, C) such that the results of the hot spot prediction (see below) should not depend on whether the prediction is based on one or the other structure.

The homology model was subjected to molecular dynamics simulations (MD) of 100 ns length in explicit water to generate a conformational ensemble for the subsequent hot spots detection. The CTD dimer remains stable during the simulation time: the RMSD of the single domains is ∼6.5 Å, and the dimer shows structural deviations of ∼8 Å (Figure S3, A in File S1). Relevant conformational changes are only observed in the region of helices H2 and H2′ (Figure 1, B) and account for most of the structural deviations observed. As such, not taking into account H2 and H2′, the RMSD of the single domains and the dimer drops to ∼3 Å (Figure S3, B in File S1). This is in agreement with previous experimental and computational findings according to which the mobility of H2 and H2′ is required for forming interactions between the CTD and substrates and, hence, Hsp90 function [33], [44].

### Hot spot prediction

**Figure 2:**
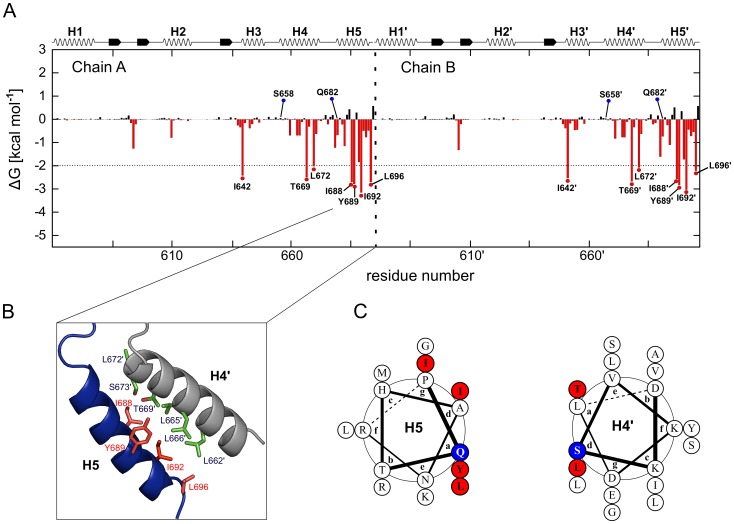
Hot spot and cold spot prediction. (A) Contribution to the dimer stabilization of each amino acid within the hHsp90 CTD. Δ*G* values are calculated by the MM-GB/SA approach [37], [83] starting from the homology model, employing a structural decomposition of the effective energy [10]. The standard error in the mean is <0.1 kcal mol^−1^ in all cases. Amino acids contributing to the dimerization with Δ*G*<−2 kcal mol^−1^ are considered hot spots and are indicated in the graphic by red dots. In addition two “cold spots” mentioned in the text are marked with blue dots. In the upper part of the panel, the secondary structure of the CTD is shown. The amino acids are numbered according to the full length hHsp90 α isoform (UniProt code: P07900). (B) Hot spot residues localized on H5 (red) and interacting residues on H4′ (green). (C) Helical wheel representation showing the position of hot spots (red) and cold spots (blue) on helices H5 and H4′.

In order to identify amino acids at the CTD interface that are crucial for dimer stability, we performed MM-GB/SA calculations combined with a decomposition of the effective energy (i.e., the sum of gas phase and solvation free energy) of dimerization on a per residue level. The approach mimics computational alanine scanning and has been applied successfully by us in retro- and prospective studies on the determinants of protein-protein interactions [10]–[12], [45]. The results reveal a distinctive interaction profile, which is almost identical for the two monomers (Figure 2, A). Residues are identified as hot spots if their contribution to the effective energy of dimerization Δ*G*<−2 kcal mol^−1^ [46].

The hot spots are spatially clustered and are located on H4 and H5, except for a single hot spot on H3. The main cluster is formed by residues I688, Y689, I692, and L696 located on H5 at the inner side of the four helix bundle (Figure 2, B). I692 and L696 form hydrophobic contacts with L662′, L665′, and L666′ located on H4′; Y689 establishes hydrophobic interactions with L665′ and L666′ (Figure 2, B) but also forms hydrogen bonds with S673′ and T669′ on H4′ and H640′ on the loop located above the N-terminal end of H3. The multiple hydrogen bond formation is possible because the side chain of Y689 adopts two conformations during the MD simulations, one where the aromatic ring points in the direction of H3′ and one where it points to H4′, i.e., the interior of the interface. In the latter case, an indentation in the binding epitope of H4′ accommodates the side chain. L672 and T669 form a second, smaller cluster on H4. The latter residue is involved in interactions with Y689′ (see above); L672 interacts with P681 on H5′. Finally, I642 on H3 is located in a peripheral position with respect to the interface but forms hydrophobic contacts with the C-terminal end of H5, that way apparently contributing to the stabilization of this secondary structure element.

As the crystal structure of an M-CTD construct of hHsp90 became available only recently (PDB code: 3Q6M) [43], we repeated the hot spot prediction for a CTD dimer of that structure, using the same settings for these computations and the prior MD simulations as in the case of the homology model. The resulting interaction profile (Figure S4 in File S1) is in very good agreement with the one of the homology model (Figure 2, A) such that all of the above mentioned hot spots are identified again. While this may have been expected from the high structural similarity between our model and the crystal structure (see above), these findings validate, in an indirect manner, the quality of our homology model and demonstrate the robustness of our MM-GB/SA-based hot spot predictions. In order to independently confirm the MM-GB/SA calculations, we performed *in silico* alanine scanning on the homology model with the DrugScore^PPI^ web server developed by us [38], [39]. The interaction profile obtained is in good agreement with the above findings, pointing to essentially the same hot spots that are crucial for hHsp90 CTD dimerization (Table S1 in File S1). We also tested if the above hot spots could have been identified by a simpler computational approach given that these hot spots are largely buried upon complex formation. For this, we computed the residue-wise relative change in the solvent-accessible surface area upon complex formation (using the SA values of the MM-GB/SA calculations starting from the CTD dimer of the crystal structure; Figure S5 in File S1). This suggests essentially all residues in the dimer interface as hot spots, indicating a pronounced loss of specificity in the predictions compared to when additional energy contributions are considered. In turn, L696, which is more peripheral to the dimer interface, is not found among the top candidates anymore when using the surface area-based approach. In our view, these findings demonstrate the predictive value of the energy-based methods.

Finally, we selected residues S658 and Q682 as “cold spots” (Δ*G* = 0.06 and −0.67 kcal mol^−1^, respectively, as calculated with the MM-GB/SA approach starting from the homology model; Figure 2, A and C), which will serve as negative controls in the subsequent experiments. These amino acids are located at the CTD interface between H5 and H4′, but they are predicted to be only marginally important for the dimerization (Figure 2, A and C). Consequently, mutating these “cold spots” to alanine should not impact the CTD stability.

### Analysis of hHsp90 stability by Thermofluor assay

**Figure 3:**
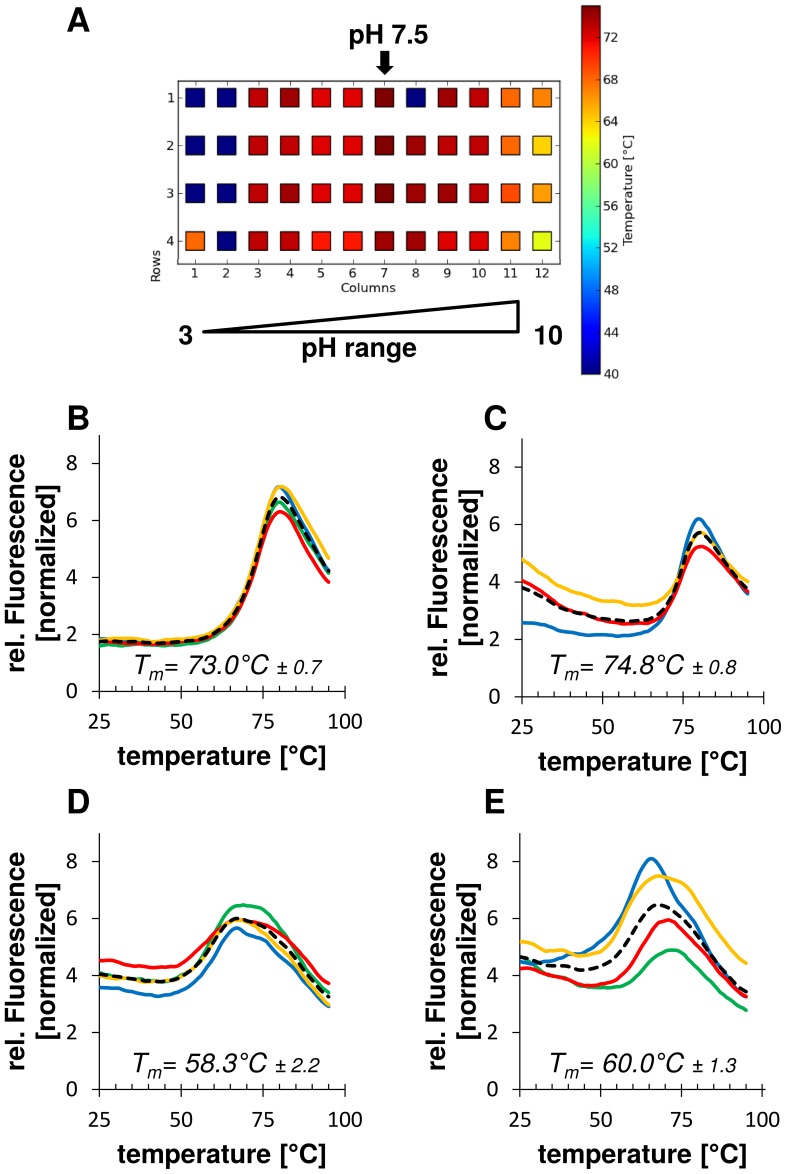
Thermofluor assay for investigating the stability of wild type and mutant hHsp90 CTD. Protein stability was analyzed in 12 different pH buffers in four independent measurements. (A) Heatmap for wild type hHsp90 CTD. Melting curves of measurements at pH 7.5 with the average *T*_m_ and standard deviation are shown below for wild type hHsp90 CTD (B), hHsp90 CTD with cold spot mutants as negative control (C), as well as hot spot alanine mutants CTD^Y689A/I692A/L696A^ (D) and CTD^I688A/Y689A/I692A^ (E). The mean value (dotted black line) was calculated from four independent measurements (yellow, red, blue, green lines) in reaction buffer with 100 mM Tris.

The stability of the CTD of hHsp90 wild type, cold spot and hot spot alanine mutants was analyzed by differential scanning fluorimetry (Table 1, Table 2) [40]. In this assay thermally-induced protein unfolding is monitored by the binding of the fluorescent dye SYPRO orange [47] to the hydrophobic core of the protein that becomes exposed upon unfolding, and the related increase in fluorescence emission. The temperature at the midpoint of the unfolding transition is defined as melting temperature (*T*_m_) of the protein [48]. A shift in *T*_m_ of a protein in its native state, or in site-specific or chemically modified forms, indicates a change in the stability of the protein [49]. In order to identify conditions under which the native state is most stable we analyzed the thermal unfolding of the purified wild type hHsp90 CTD in the pH range from 3–10. These studies (Figure 3, A) showed that the CTD of hHsp90 is most stable at mild neutral conditions (pH 7.5). Thus, screening of all hot spot and cold spot variants of hHsp90 was done at this pH condition.

**Table 1:** Variants of the CTD of hHsp90 investigated in this study.

Variant	Abbreviation	MW[a]	Extinction coefficient
Wild type	wt	21469.3	13075
Cold spot mutant	CTD^S658A/Q682A^	21396.2	13075
Hot spot mutant I	CTD^Y689A/I692A/L696A^	21293.0	11585
Hot spot mutant II	CTD^I688A/Y689A/I692A^	21293.0	11585

[a] Computed molecular weight in Da.

**Table 2:** *T*_m_ of hHsp90 CTD wild type and alanine mutants.

	CTD wt	CTD^S658A/Q682A^	CTD^Y689A/I692A/L696A^	CTD^I688A/Y689A/I692A^
*T*_m_ [a]	73.0±0.7	74.8±0.8	58.3±2.2	60.0±1.3
Δ*T*_m_ [b]	0.0	1.8	−14.8	−13.0

[a] The detected fluorescence signal corresponds to the denaturation state of hHsp90. The melting temperature *T*_m_ of hHsp90 CTD wild type, cold spot, and alanine mutants was determined from the derivative of the fluorescence data by the implemented software (qPCRsoft V2.0.37.0, Analytik Jena AG, Germany). The mean value and standard deviation were calculated from four independent measurements in reaction buffer with 100 mM Tris at pH 7.5 in °C.

[b] Difference in the *T*_m_ with respect to the wild type in °C.

The wild type form of hHsp90 is characterized by a melting temperature of 73°C (Figure 3, B). In contrast, hot spot substitution mutants carrying alanines at positions 689, 692 and 696 (CTD^Y689A/I692A/L696A^, Figure 3, D) or at positions 688, 689 and 692 (CTD^I688A/Y689A/I692A^, Figure 3, E) show a significant decrease (ΔT_m_≥13°C) in their melting temperatures indicating a substantial loss in the stability due to the substitution of the native hHsp90 residues in these positions by the small, non-polar alanine. These triple mutations were chosen, respectively, out of the four hot spots of the main cluster because they result in patterns of sequence localization that could be mimicked by non-peptidic α-helix mimetics (see Discussion for further details).

Additionally, alanine single mutants were analyzed at the same conditions to reveal the potential contribution of individual positions at the dimerization interface on the stability of the CTD dimer. To this end we substituted the predicted hot spots at positions I688, Y689, I692 and L696 individually to alanines (Table S2, Table S3 in File S1) and determined the melting temperature and protein stability of the single mutants. Compared to the alanine triple mutants, all of the single mutants showed a much lower reduction (ΔT_m_<8°C) in their melting temperature with respect to the wild type of hHsp90 (Figure S6, Table S4 in File S1). Alanine substitutions at positions S658 and Q682 identified as cold spots (Figure 2, A–C) had no effect on the stability of the protein. The related mutant (Figure 3, C) showed a melting temperature corresponding to the hHsp90 wild type.

### Analysis of hHsp90 multimer stability by size exclusion chromatography

**Figure 4:**
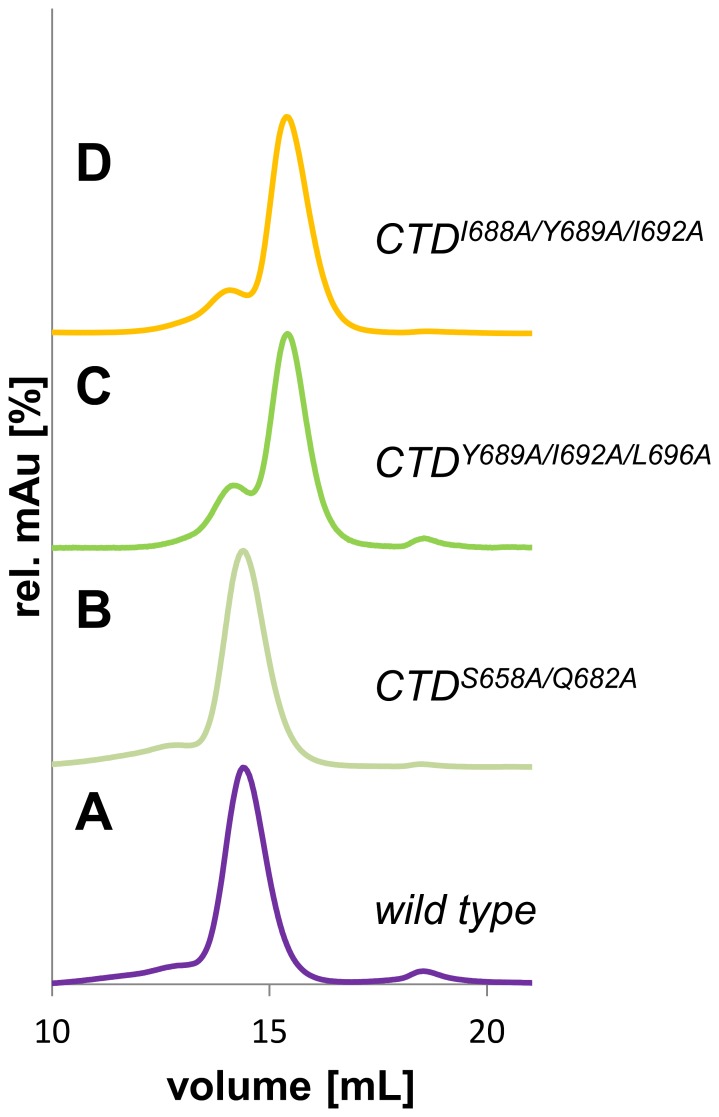
Elution profiles of hHsp90 CTD variants using size exclusion chromatography. Chromatograms for wild type hHsp90 CTD (A), cold spot (B), and hot spot alanine mutants (C and D). Experiments were performed in triplicates on a Superdex SD200 10/300 column in HPLC-buffer (10 mM MES/KOH, 200 mM KCl, 1 mM EDTA, 1% Glycerol) at pH 6 with 110 µL of purified hHsp90 CTD. The molecular weight of hHsp90 CTD variants was calculated based on the slope of the calibration curve obtained with standard proteins. The elution peak of the wild type corresponds to a molecular weight of 88±0.5 kDa, indicating an apparent tetrameric complex. The cold spot mutant shows the same elution profile as the wild type protein corresponding to a molecular weight of 88±0.3 kDa. Alanine mutants CTD^Y689A/I692A/L696A^ and CTD^I688A/Y689A/I692A^ show a shift to lower molecular weights of 57–58 kDa (57±0.2 kDa for CTD^Y689A/I692A/L696A^ and 57±0.4 kDa for CTD^I688A/Y689A/I692A^). This indicates a smaller protein complex suggesting a weakly associated dimer or closely associated trimer configuration.

The effect of alanine substitutions in predicted hot and cold spots positions of hHsp90 with respect to disrupting the oligomerization state of the protein was analyzed by SEC. In order to relate the elution volume on the gel filtration column to the molecular mass of the purified wild type and alanine mutants of hHsp90 (Table 1), a number of protein standards (Lysozyme 14.000 Da, Carbonic Anhydrase 29.000 Da, BSA 66.000 Da, Alcohol Dehydrogenase 150.000 Da, β-Amylase 200.000 Da, Apoferritin 443.000 Da) were applied to the gel filtration column. Based on the calibration proteins the main peak of the elution profile (at a buffer volume of ∼14 mL) of the wild type CTD corresponds to a molecular mass (Mw) of 80–90 kDa. Assuming that the overall fold of the recombinant CTD resembles the globular form of the protein standards, the Mw calculated from the SEC experiments implies that the wild type CTD exists as tetramer in solution (Figure 4, A). However, if the protein analyzed by SEC is asymmetrical or elongated - a condition met with the CTD of hHsp90 - the protein can easily elute at a position twice the Mw of a globular protein [50]. Thus, the apparent Mw calculated from the SEC experiments and the complex stoichiometry deduced from this should not be taken as absolute numbers in the case of the hHSp90 CTD but rather as relative measures to indicate whether mutations at the dimerization interface affect the stability of the complex. At a lower elution volume of ∼13 mL, a small shoulder is visible in the elution profile that, based on the calibration, corresponds to Mw = 160–190 kDa, i.e., an apparent octamer. Figure 4, C–D shows the elution profile for the hot spot mutants CTD^Y689A/I692A/L696A^ and CTD^I688A/Y689A/I692A^. Compared to the wild type the main fractions of both hHsp90 mutants elute at higher buffer volumes (∼15.5 mL) from the column. This clearly shows that these variants predominantly possess a lower molecular mass than the wild type and form complexes of lower subunit stoichiometry. Based on the calibration with the globular protein standards each of the alanine substitution mutants has a molecular mass of 58 kDa, which would indicate an apparent trimeric or dimeric state. In addition, a smaller peak is visible in the elution profile at a buffer volume similar to that found for the wild type, indicating a low population of apparent tetramers. In contrast, the elution profile of the cold spot mutant was virtually identical to that of the CTD of wild type hHsp90 (Figure 4, B): The main fraction of the mutant eluted at a buffer volume comparable to that of the main peak of hHsp90 wild type, with a small shoulder visible again at a lower buffer volume. This indicates that the molecular masses of both variants are comparable. Taking together the information of the different gel filtrations, we conclude that the organizational state of the CTD of hHsp90 is larger in the wild type and the cold spot variants, whereas the hot spot variants predominantly form complexes of lower stoichiometry. The data clearly indicate that the hot spot mutations substantially affect the apparent Mw and the complex stoichiometry.

### Multi-angle light scattering

**Figure 5:**
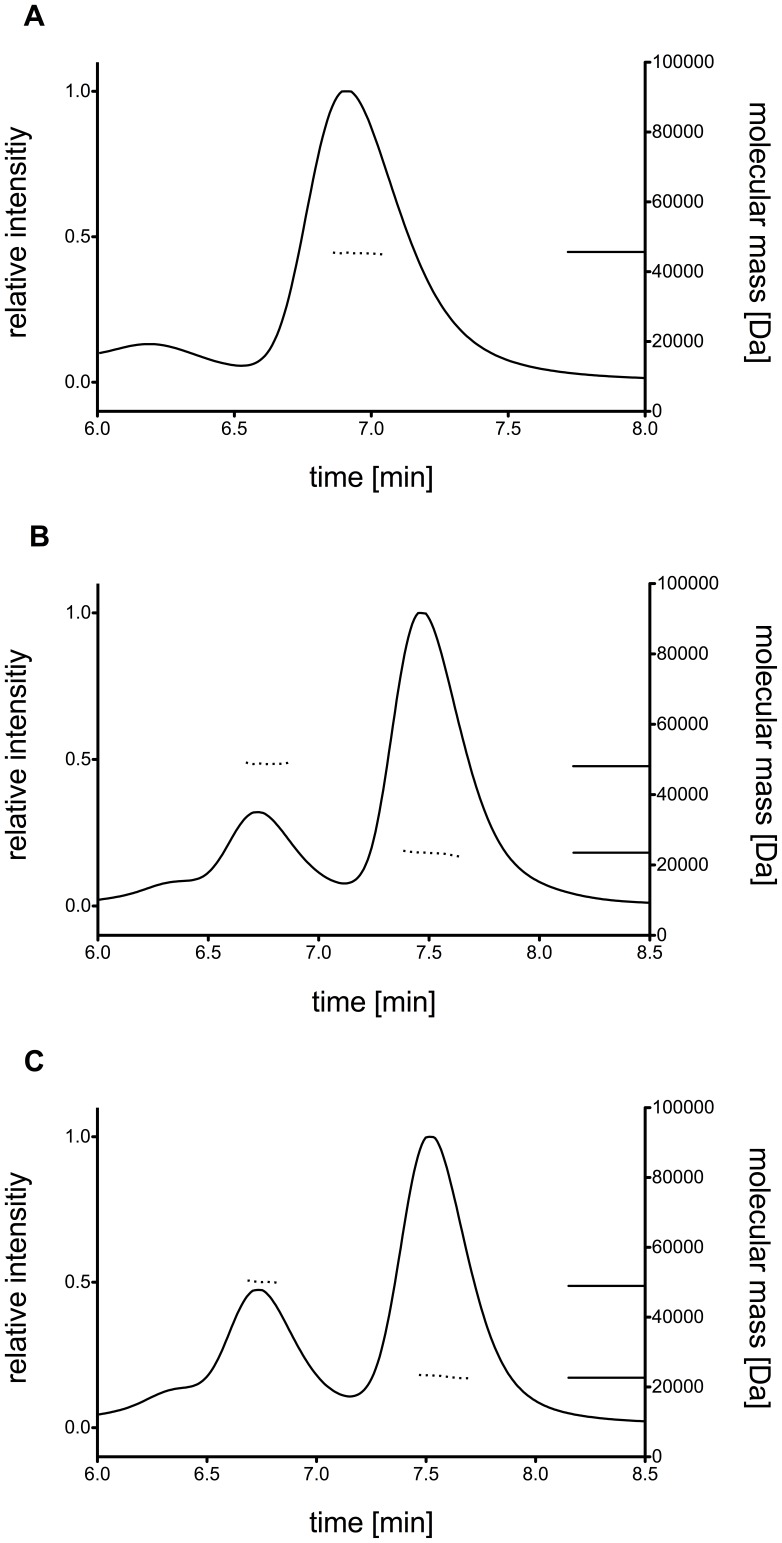
Differential refractive index and molecular mass of hHsp90 variants using multi-angle light-scattering. (A) For wild type hHsp90 CTD a species with molar mass of 45.4±0.1 kDa was determined. Molar masses of higher oligomeric species could not be specified due to insufficient separation. (B, C) Hot spot mutants of hHsp90 CTD, CTD^Y689A/I692A/L696A^ (B) and CTD^I688A/Y689A/I692A^ (C), revealed species with molar masses of 23.5±0.2 kDa and 23.2±0.2 kDa, respectively, and additional species with molar masses of 48.7±0.5 kDa and 50.1±0.5 kDa, respectively. Furthermore, also higher oligomeric species were detectable but could not be analyzed with respect to molar masses.

In order to resolve whether the effects of the hot spot mutations on the apparent Mw correspond to a shift from a tetramer to a loosely associated dimer or from an elongated dimer to a monomer, we performed MALS experiments on wild type CTD and the two hot spot mutants. MALS allows determining the absolute molar mass of particles in solution. For the wild type, the experiments revealed a predominant species of 45.4±0.1 kDa (Table 3; Figure 5, A), in almost perfect agreement with the expected Mw of 43 kDa of the dimer CTD (Table 1). Additionally, higher oligomeric species are present eluting earlier from the column (Figure 5, A). Although we could not clearly assign a specific mass for this peak and thereby the exact oligomeric state due to a low population and an insufficient separation from the dimer signal, this species might represent a tetramer. For both hot spot mutants CTD^Y689A/I692A/L696A^ and CTD^I688A/Y689A/I692A^, the predominant species detected had Mw = 23.5±0.2 and 23.2±0.2 kDa, respectively (Table 3; Figure 5, B and C), corresponding to monomeric CTDs (Table 1). In addition, with a population of ∼23% and ∼31%, respectively, species with Mw = 48.7±0.5 kDa and Mw = 50.1±0.5 kDa were detected, corresponding to residual dimeric CTDs. With a much lower population, higher oligomeric species were detected again (Figure 5, B and C). Taken together, the MALS experiments reveal that hot spot mutations substantially influence the stability of the CTD complex in that the predominant form of the wild type CTD is a dimer whereas the predominant forms of the hot spot mutants are monomers.

**Table 3:** Molecular weights and mass distributions measured by MALS.

	Mass species 1 [a]	Mass species 2 [a]	Distribution species 1/species 2
CTD wt	-	45.4±0.1	0/1
CTD^Y689A/I692A/L696A^	23.5±0.2	48.7±0.5	0.77/0.23
CTD^I688A/Y689A/I692A^	23.2±0.2	50.1±0.5	0.69/0.31

[a] In kDa.

### Circular dichroism spectroscopy

To demonstrate that the predicted hot spots have no impact on the overall protein folding, circular dichroism (CD) spectroscopy measurements were performed. We observed similar spectra for wild type, hot spot, and cold spot mutants (Figure S7 in File S1) with minima at 207 nm and 225 nm that indicate a predominately α-helical secondary structure of hHsp90 CTD, which agrees well with the secondary structure content derived from the CTD of the crystal structure (data not shown). Similar to the hot spot and cold spot mutants do the alanine single mutants show a high α-helical secondary structure (Figure S7 in File S1). Taken together, the CD measurements underline that the selected substitutions in the hot spot, cold spot, and single mutants do not perturb the overall folding of the hHsp90 CTD.

## Discussion

Identifying hot spots in protein-protein interfaces yields insights into the energetics of PPIs that can be exploited for the identification of PPI modulators [10]–[12], [45], [51]. Here, we aimed at identifying hot spot residues that determine the stability of the hHsp90 CTD dimer following the idea that inhibiting CTD dimerization should provide a novel way to therapeutically interfere with Hsp90 activity. Performing MM-GB/SA calculations together with a structural decomposition of the effective binding energy [10], we identified a main cluster of four hot spot residues (I688, Y689, I692 and L696) located on H5 in the dimer interface. The importance of these residues for dimerization was also confirmed by *in silico* alanine scanning [38], [39]. A smaller cluster of two residues (T669 and I672) and a single hot spot (I642) were predicted by these methods, too. The residues in the main cluster have a mainly hydrophobic character, leading to multiple hydrophobic contacts of these residues with residues on H4′. In addition, Y689 forms hydrogen bond interactions with several residues on H4′. Residues that are able to form hydrophobic and stacking interactions and at the same time engage in polar interactions such as tyrosine, arginine, and tryptophan are frequently found as hot spots in protein-protein interfaces [52]. The influence of the hot spot residues in the main cluster on the stability of the hHsp90 CTD was experimentally confirmed by SEC, MALS, and differential scanning fluorimetry. In contrast to the wild type, for which the CTD dimer is the predominant form in solution, monomeric forms are the predominant species of the CTD^I688A/Y689A/I692A^ and CTD^Y689A/I692A/L696A^ mutants; in addition, a pronounced decrease of the melting temperature by more than 13°C was found for these mutants compared to the wild type. The reduced subunit stoichiometry and the loss of stability indicate that the interaction between individual monomers in the complex is disrupted by substituting the hot spot clusters Y689/I692/L696 or I688/Y689/I692 with small, nonpolar alanine residues. Data on all four single alanine mutants demonstrate that each substitution at a single position destabilizes the CTD dimer, too, albeit to only half of the extent observed for the triple mutants. This suggests that the destabilizing effects of single mutations at the predicted hot spot positions are additive.

In previous studies, a dimer was identified as the basic functional unit of hHsp90 [33], [53]. In this work, analysis of the Mw of the CTD by SEC and calibration with protein standards suggests that the isolated hHsp90 CTD exists as an apparent tetramer under physiological conditions, although a dimer might better describe the stoichiometry of the complex in solution when the asymmetrical, elongated form of the CTD is kept in mind. Our MALS experiments demonstrate that the predominant form of wild type CTD is a dimer. Our SEC experiments furthermore show that complex formation in the CTD significantly depends on a few hot spot positions at the dimerization interface of the four-helix bundle (I688, Y689, I692 and L696): Substitution of these residues results in a decomposition of the complex into monomers, as shown by our MALS experiments. Other residues at the same interface play a less pivotal role in the stabilization of the CTD dimer: Substitution of these positions to alanine has no effect on the stoichiometry of the hHsp90 CTD complex as shown for the S658A/Q682A cold spot mutant, which resulted in an elution profile in our SEC studies essentially undistinguishable from that of the wild type CTD.

In both the SEC and MALS experiments low populations of higher oligomeric species of wild type CTD were observed, with the SEC experiments suggesting a species with twice the Mw as the predominant dimeric form. The ability of Hsp90 to self-oligomerize has been described before [54], [55], and tetrameric and higher even-numbered species have been found [55]. Structural information on a hexameric assembly has been obtained for the isolated hHsp90 M-CTD (aa 293–732) in recent crystallization studies [43]; these crystals revealed that M provides the essential contacts for the hexameric arrangement in this construct. Hence, while electrophoretic studies of deletion mutants of Hsp90 also demonstrated that the C-terminal 200 amino acids are able to form oligomers [54], it is still not clear which residues form the interface mediating the potential tetrameric assembly in our isolated CTD.

The information on the complex stability of the wild type, the hot spot, and cold spot mutants obtained from our SEC and MALS studies is confirmed by differential scanning fluorimetry. Although the Thermofluor assay does not directly detect dissociation and stoichiometry of the CTD complex in the different hHsp90 variants, the decreased melting temperatures observed for the CTD hot spot mutants correlate well with their expected role in stabilizing the interaction at the four-helix bundle interface and, thereby, the interaction of the entire complex. As the melting temperature reflects the transition of a protein from its native to the denatured state, a reduction of the melting temperature as observed for the hot spot mutants is indicative of a less stable protein or protein assembly. Analysis of protein stability by Thermofluor assay has been already successfully applied in previous studies to resolve different thermostabilities of wild type and mutant proteins [56], [57]. As for the hHsp90, hot spot mutants reduced melting temperatures were observed for the mutants of the MMACHC protein [58]. However, the high melting temperature of 73°C observed in our study for the hHsp90 wild type is rather untypical for proteins studied by Thermofluor analysis, which usually show much lower melting temperatures. Hence, the high melting temperature observed with the hHsp90 CTD in our study probably reflects the molecular function of Hsp90 to serve as thermo-tolerant molecular chaperone [59]. Similar to the unusual melting temperature itself, hHsp90 CTD wild type and hot spot mutants showed a large difference in their melting temperature (Δ*T*_m_≥13°C). Thermofluor studies on other proteins showed a less pronounced effect (Δ*T*_m_<5°C) of mutations on the melting temperatures [58], [60]. Hence, the large differences observed for wild type and hot spot mutants of hHsp90 are clearly indicative that the positions at the dimerization interface identified by our computational approach have a substantial effect on the stability of the protein complex. Such an influence of interface interactions has been described previously for a variety of protein systems [45], [61]–[64].

Cimmperman *et al.* [65] showed that the melting temperature of a protein is also controlled by ligand binding. As the C-terminal domain of Hsp90 was proposed to have an ATP binding site, too, in addition to the one in the N-terminal domain [35], [66] we have studied the effect of the ligands Mg and ATP on the stability of the hHsp90 CTD by Thermofluor analysis. We observed an increase in the melting temperature by 2°C when one of the ligands was present (Table S5 and Figure S8 in File S1). When compared to the high difference obtained with the hot spot mutants this quantity again underlines the large influence of the selected point mutations at the dimerization interface on the stability of the protein complex.

Mimicking localized interactions in hot spot regions by small molecules has been found as a viable way to interfere with PPIs [4], [11], [12], [67]–[69]. Regarding the physicochemical properties of our hot spots, the predominantly hydrophobic character is in line with the rule of four [70], a characterization of the chemical space of PPI inhibitors, which suggests that inhibitors should have log P>4. At the same time, inhibitors should be able to form polar interactions, which confer specificity of binding [71]; this would be given when mimicking Y689. The latter is also of interest because Y689 is accommodated by an indentation in the binding epitope of H4′. In this context, PPI inhibitors have been found to be particularly effective when they bind to well-defined clefts or grooves in the protein-protein interface [7], [72]. Finally, the hot spots in the main cluster show an *i*, *i*+4, *i*+8 pattern with respect to sequence localization when starting with I688, which could be mimicked by a novel class of non-peptidic α-helix mimetics recently described [73]. In reverse order, an *i*, *i*+4, *i*+7 pattern emerges, for which several scaffolds for α-helix mimetics have been described [74], [75]. Thus, it seems promising to use the hot spots of the main cluster as a pharmacophoric template [12] for searching and designing inhibitors that interfere with the dimerization of hHsp90.

In summary, our computational results reveal the presence of spatially clustered hot spot residues in the hHsp90 CTD interface, which form a functional epitope and account for most of the protein dimerization energy. The influence of these residues on the stability and the oligomeric state of the CTD has been demonstrated by experiments. These hot spots have favorable properties with respect to using them as a pharmacophoric template for identifying and designing small-molecule inhibitors of hHsp90 dimerization. This opens up a new avenue for interfering with hHsp90 function for treating cancer.

## Materials and Methods

### Materials

Chemicals and reagents were purchased from AppliChem, Sigma-Aldrich, Carl Roth, VWR, Merck and Fluka at analytical grade. Plasmids were derived from pET vectors from Merck/Novagen (Darmstadt, Germany). SYPRO Orange was obtained from Sigma-Aldrich (Steinheim, Germany).

### Multiple sequence alignment and homology model

Sequences of the Hsp90 CTD from *S. cerevisiae* and *E. coli* were retrieved from the Protein Data Bank (PDB). Two sequences from *S. cerevisiae* were used, corresponding to the PDB entries 2CG9 and 2CGE (UniProt code P02829, amino acids 540–677). One sequence from *E. coli* was considered, corresponding to the PDB entry 1SF8 (UniProt code P0A6Z3, amino acids 510–624). The sequence of the hHsp90 α isoform CTD was retrieved from the UniProt database (UniProt code P07900, amino acids 561–697). A multiple sequence alignment was generated by means of CLUSTALW [76], [77].

Five homology models for the hHsp90 CTD were developed using the “automodel” procedure and default parameters in MODELLER 9.8 [41]. As templates, crystal structures from *S. cerevisiae* (PDB codes 2CG9 and 2CGE) and *E. coli* (PDB code 1SF8) were used. The best model as evaluated from the MODELLER objective function was chosen for the subsequent work. The quality of this homology model was assessed by means of PROCHEK [42] using default parameters.

### Molecular dynamics simulations

All the procedures described in the following were performed with the Amber 11 software package [78], using the ff99SB force field [79]. The homology model and a CTD dimer of a crystal structure of an M-CTD construct of hHsp90 (PDB code: 3Q6M) were initially placed in an octahedral periodical box of TIP3P water molecules [80], where the distance between the edges of the box and the closest atom of the solute is at least 11 Å. Long-range electrostatic interactions were treated using the Particle Mesh Ewald (PME) method [81], and the SHAKE algorithm [82] was employed to constrain bond lengths of heavy atoms to hydrogen atoms. The time step for all MD simulations was set to 2 fs, with a non-bonded cutoff of 8 Å. The homology model and the CTD dimer of the crystal structure, first, were geometry-optimized by 10 rounds of energy minimization; in each round 50 steps of steepest descent minimization were followed by 450 steps of conjugate gradient minimization, applying decreasing harmonic restraints on the solute atoms (the force constant was 25 kcal mol^−1^ Å^−2^ in the first five rounds, and reduced to 5 kcal mol^−1^ Å^−2^ in the remaining). The systems were heated from 100 K to 300 K during an MD simulation of 50 ps length performed in the canonical (NVT) ensemble, applying harmonic restraints with force constants of 5 kcal mol^−1^ Å^−2^ to all solute atoms. Afterwards, MD simulations of 250 ps length in the isothermal-isobaric ensemble (NPT) were performed applying the same harmonic restraints as in the previous step, in order to adjust the solvent density. Finally, MD simulations of 100 ps length in the NVT ensemble were performed, gradually reducing the force constants of the harmonic restraints on the solute atoms to zero. Additional 100 ns of MD simulations at 300 K were performed, and the coordinates were stored every 20 ps. These were used to extract 5000 snapshots for calculating the effective energy of dimerization and the structural decomposition.

### MM-GB/SA calculations, free energy decomposition and in silico alanine scanning

MM-GB/SA calculations were performed employing the “single trajectory approach” [83]. All counterions and water molecules were stripped. For each snapshot, the gas-phase energy (i.e., the sum of the internal energies plus electrostatic and van der Waals energies) was calculated based on the ff99SB force field [79] without applying a non-bonded cutoff. Polar contributions to the solvation free energy were calculated using the “OBC” generalized Born model [84] together with mbondi2 radii, with a dielectric constant of 1 for the solute and 80 for the solvent. The polar contributions were computed at 100 mM ionic strength. Nonpolar contributions to the solvation free energies were calculated by a solvent-accessible surface area (SASA)-dependent term using a surface tension of γ = 0.0072 kcal mol^−1^ Å^−2^. Changes in the configurational entropy upon dimerization were not considered. The contributions on a per-residue basis to the overall effective energy (i.e., sum of gas-phase plus solvation free energy) of dimerization were calculated employing the decomposition scheme implemented in Amber 11 [78]. The same snapshots were used to perform *in silico* alanine scanning using the DrugScore^PPI^ web server [38], [39].

### Cloning, expression and purification

Synthetic codon optimized DNA (GeneScript, Piscataway, NJ) corresponding to the coding region of residues 563–732 of hHsp90 was cloned into expression vector pTEV21-a in *E. coli* BL21 (DE3) (Agilent Technologies). Cultures were grown at 37°C in LB media (5 g l^−1^sodium chloride, 5 g l^−1^ yeast extract and 10 g l^−1^ peptone) with ampicillin to OD_600_ 0.8–1.2. The production of recombinant protein was induced by adding 1 mM isopropyl-1-thio-*β*-D-galactopyranoside, and cells were grown for another 4 h at 28°C. Cells were harvested by centrifugation, suspended in binding buffer (40 mM HEPES, 20 mM KCl, 1 mM DTT, 1 mM EDTA, 0.002% PMSF, pH 7.5) [85] and disrupted by sonification. Recombinant proteins were purified via a C-terminal His_6_-tag by immobilized metal ion affinity chromatography to homogeneity (Figure S9 in File S1).

### Alanine mutants and cold spot creation

Alanines at positions 689, 692, and 696 as well as at positions 688, 689, and 692 for the hot spot mutants and at positions 658 and 682 for the cold spot mutant were introduced into the constructs as described in the QuikChange Site-Directed Mutagenesis Kit (Stratagene) using *Pwo* polymerase and designed mutagenesis primers (Table 4). Additionally, alanine single mutants were constructed with alanines at positions 688, 689, 692, and 696, respectively (Table S3 in File S1).

**Table 4:** Mutagenesis primers[a].

**Cold spot mutant**: CTD^S658A/Q682A^
Primer 1: TCG (Ser) → GCA (Ala): 45 nt (5′-3′)
*Forw*.: CAGAAGCTGATAAAAACGACAAA**GCA**GTGAAAGATCTGGTTATCC
*Rev*.: GGATAACCAGATCTTTCAC**TGC**TTTGTCGTTTTTATCAGCTTCTG
Primer 2: CAG (Gln) → GCA (Ala); 34 nt (5′-3′)
*Forw*.: AGCCTGGAAGACCCG**GCA**ACGCATGCCAACCGTA
*Rev*.: TACGGTTGGCATGCGT**TGC**CGGGTCTTCCAGGCT
**Hot spot mutant I**: CTD^Y689A/I692A/L696A^
TAC (Tyr) → GCA (Ala); ATC (Ile) → GCA (Ala); CTG (Leu) → GCA (Ala): 44 nt (5′-3′)
*Forw*.: CAACCGTATT**GCA**CGCATG**GCA**AAACTGGGC**GCA**GGTATTGATG
*Rev*.: CATCAATACC**TGC**GCCCAGTTT**TGC**CATGCG**TGC**AATACGGTTG
**Hot spot mutant I**: CTD^I688A/Y689A/I692A^
ATT (Ile) → GCA (Ala); TAC (Tyr) → GCA (Ala); ATC (Ile) → GCA (Ala): 37 nt (5′-3′)
*Forw*: GCATGCCAACCGT**GCAGCA**CGCATG**GCA**AAACTGGGCC
*Rev*: GGCCCAGTTT**TGC**CATGCG**TGCTGC**ACGGTTGGCATGC

[a] The primers were obtained by Sigma-Aldrich Chemie GmbH Steinheim, Germany. Bold nucleotides indicate the newly introduced alanines.

### Stability assay by Fluorescence Thermal Shift Assay (Thermofluor)

Stability assay was performed in the real-time thermo-cycler qTOWER 2.0 (Analytik Jena AG, Germany) with 0.15 mg/mL protein and the fluorescent dye SYPRO Orange (1∶1000) in 96 well PCR plates. The fluorescence signal of the SYPRO Orange dye was measured at an initial start temperature of 25°C. Up to 12 different conditions were tested (Table 5) [86] with increasing the temperature stepwise up to 95°C. When hydrophobic residues of the protein become more accessible, this leads to binding of SYPRO Orange. Fluorescence changes in the wells of the plate were monitored simultaneously with Channel Photo Multiplier (CPM). The wavelengths for excitation and emission were 490 nm and 580 nm, respectively. The detected fluorescent signal corresponds to the denaturation state of hHsp90. Melting points were determined by the implemented software (qPCRsoft V2.0.37.0, Analytik Jena AG, Germany) from the derivative of the fluorescence data.

**Table 5:** pH values of screening buffers [86] for Thermofluor assay.

No.	Buffer [100 mM]	pH
1	Glycine	3.0
2	Citric acid	4.0
3	Sodium citrate	5.5
4	Sodium/potassium phosphate	6.0
5	MES	6.2
6	Bis-tris propane	6.5
7	Sodium/potassium phosphate	7.0
8	Tris	7.5
9	EPPS	8.0
10	Tris	8.5
11	CHES	9.0
12	CHAPS	10.0

### Size exclusion chromatography

Size exclusion chromatography analyses were accomplished in triplicates on a Superdex SD200 10/300 column on an ÄKTAprime plus chromatography system (GE lifescience). The experiments were performed at 4°C in HPLC buffer (10 mM MES, 200 mM KCl, 1 mM EDTA, 1% Glycerol) with a flow rate of 0.5 mL/min at pH 6. Samples were centrifuged before for 15 min at 14.000xg, and 110 µL of the purified CTD of hHsp90 was loaded on the column. Data were analyzed using the software PrimeView Evaluation 5.0 (UNICORN, GE), and the maximal absorbance was determined by peak integration.

### Multi-angle light scattering

A size exclusion chromatography (SEC) column (Bio SEC-5, 150 Å, Agilant Technologies) was equilibrated with the above HPLC buffer without glycerol using a HPLC system (Agilant Technologies) connected with a multi-angle light-scattering detector (miniDAWN TREOS, Wyatt Technologies) and a differential refractive-index detector (Optilab T-rEX, Wyatt Technologies). Samples with concentrations of 2.4 mg/ml were centrifuged at 74.000xg for 30 minutes at 4°C and loaded onto the equilibrated SEC column and data were analyzed with the ASTRA software (Wyatt Technologies).

### CD spectroscopy

CD measurements for wild type hHsp90 CTD as well as for all mutants generated (CTD^I688A/Y689A/I692A^, CTD^Y689A/I692A/L696A^, CTD^S658A/Q682A^, CTD^I688A^, CTD^Y689A^, CTD^I692A^, and CTD^L696A^) were performed on a Jasco J-715 spectrometer, using 0.2 mg/ml protein solutions in 50 mM potassium phosphate buffer, pH 7.6 and a cuvette with a path length of 1 mm. The spectra were obtained by averaging five measurements for each protein sample and correcting the signal by subtracting the buffer signal. Data points were collected every 0.2 nm in the range from 195 to 260 nm.

## Supporting Information

File S1 **The file contains additional information to the manuscript explaining results in further details.** It consists of 14 pages, 5 tables and 9 figures.
(PDF)
