# Supplementary material for: Resolving Hot Spots in the C-Terminal Dimerization Domain that Determine the Stability of the Molecular Chaperone Hsp90
Source: PLoS One. 2014 Apr 23;9(4):e96031. doi: 10.1371/journal.pone.0096031 (PMC3997499; doi:10.1371/journal.pone.0096031)
Supplement: File S1 — The file contains additional information to the manuscript explaining results in further details. It consists of 14 pages, 5 tables and 9 figures. (PDF) [file pone.0096031.s001.pdf]

Supporting Information

Resolving Hot Spots in the C-Terminal Dimerization Domain that Determine the Stability of the Molecular Chaperone Hsp90

Emanuele Ciglia[1§], Janina Vergin[2§], Sven Reimann[3], Sander H.J. Smits[3], Lutz Schmitt[3], Georg Groth[2], and Holger Gohlke*[1]

[1]Institute for Pharmaceutical and Medicinal Chemistry, Heinrich-Heine-University, Düsseldorf, Germany

[2]Institute for Biochemical Plant Physiology, Heinrich-Heine-University, Düsseldorf, Germany

[3]Institute of Biochemistry, Heinrich-Heine-University, Düsseldorf, Germany

[§]These authors contributed equally to this work.

[*]To whom correspondence should be addressed: Universitätsstr. 1, 40225 Düsseldorf, Germany, Phone: +49-211-81-13662, Fax: +49-211-81-13847, Email: gohlke@uni-duesseldorf.de

Supplemental Tables

Comparison of hot spot predictions with MM-GB/SA and DrugScore[PPI]

Table S1: Predicted hot spot residues of the hHsp90 CTD.[a]

Residue	MM-GBSA ΔG[b]	DrugScore[PPI] $\Delta\Delta G$[c]
I642	-2.52	-1.85
T669	-2.66	-0.41
L672	-2.04	-0.92
I688	-2.63	-1.23
Y689	-2.80	-1.52
I692	-3.20	-1.57
L696	-2.50	-0.81

[a] In kcal mol^{-1}.

[b] Mean values of effective energy contributions to the dimerization of hHsp90 CTD as computed with MM-GB/SA calculations starting from the homology model [1]. The standard error in the mean is < 0.1 kcal mol^{-1}.

[c] *In silico* alanine scanning results with DrugScore[PPI] [2].

hHsp90 CTD single alanine mutants

Table S2: Single alanine mutants of the CTD of hHsp90 investigated in this study.

Variant	Abbreviation	MW[a]	Extinction coefficient
Alanine mutant I	CTD^{I688A}	21427.2	13075
Alanine mutant II	CTD^{Y689A}	21377.3	11585
Alanine mutant III	CTD^{I682A}	21427.2	13075
Alanine mutant IV	CTD^{L696A}	21427.2	13075

[a] Computed molecular weight in Da.

Table S3: Mutagenesis primers for single alanine mutants.[a]

**I688A:**<br>ATT (Ile) → GCA (Ala): 30 nt (5'-3')<br>*Forw.*: CATGCCAACCGT**GCA**TACCGCATGATCAAA<br>*Rev.*: TTTGATCATGCGGTA**TGC**ACGGTTGGCATG
**Y689A:**<br>TAC (Tyr) → GCG (Ala): 31 nt (5'-3')<br>*Forw.*: ATGCCAACCGTATT**GCG**CGCATGATCAAACT<br>*Rev.*: AGTTTGATCATGCG**CGC**AATACGGTTGGCAT
**I692A:**<br>ATC (Ile) → GCG (Ala): 30 nt (5'-3')<br>*Forw.*: ATTTACCGCATG**GCG**AAACTGGGCCTGGGT<br>*Rev.*: ACCCAGGCCCAGTTT**CGC**CATGCGGTAAAT
**L696A:**<br>CTG (Leu) → GCG (Ala): 31 nt (5'-3')<br>*Forw*: ATCAAACTGGGC**GCG**GGTATTGATGAAGATG<br>*Rev*: CATCTTCATCAATACC**CGC**GCCCAGTTTGAT

[a] The primers were obtained by Sigma-Aldrich Chemie GmbH Steinheim, Germany. Bold nucleotides indicate the newly introduced alanines.

Table S4: T_m of hHsp90 CTD wild type and single alanine mutants.

	CTD wt	CTD^{I688A}	CTD^{Y689A}	CTD^{I692A}	CTD^{L696A}
T_m[a]	73.0 ± 0.7	66.7 ± 1.8	65.4 ± 0.9	66.1± 1.3	65.1 ± 0.7
ΔT_m[b]	0.0	-6.3	-7.6	-6.9	-7.9

[a] The detected fluorescence signal corresponds to the denaturation state of hHsp90. The melting temperature T_m of hHsp90 CTD single alanine mutants was determined from the derivative of the fluorescence data by the implemented software (qPCRsoft V2.0.37.0, Analytik Jena AG, Germany). The mean value and standard deviation were calculated from at least three independent measurements in reaction buffer with 100 mM Tris at pH 7.5 in °C.

[b] Difference in the T_m with respect to the wild type in °C.

Thermofluor analysis of hHsp90 CTD with ligands

Table S5: *T*m of hHsp90 wild type in the presence of ATP or MgCl2.

	pH 7	pH 7.5	pH 8	pH 8.5
wt T_m[a]	73.5±0.5	73.0±0.7	71.5±1.1	71.8±0.4
ATP T_m[a]	75.5±0.9	74.8±0.4	73.8±0.4	73.5±1.1
ΔT_m ATP[b]	+2.0	+1.3	+1.5	+2.3
$MgCl_2$ T_m[a]	75.0±0.0	75.0±0.7	74.5±0.5	73.8±0.8
ΔT_m $MgCl_2$ [b]	+1.5	+1.5	+2.3	+2.3

[a] The detected fluorescence signal corresponds to the denaturation state of hHsp90. The melting temperature T_m of hHsp90 CTD wild type in the presence of ATP or $MgCl_2$ was determined from the derivative of the fluorescence data by the implemented software (qPCRsoft V2.0.37.0, Analytik Jena AG, Germany). The mean value and standard deviation were calculated from four independent measurements in reaction buffer with 100 mM Tris at pH 7, 7.5 8 and 8.5 in °C.

[b] Difference in the T_m with respect to the wild type in °C.

Supplemental Figures

Sequence alignment

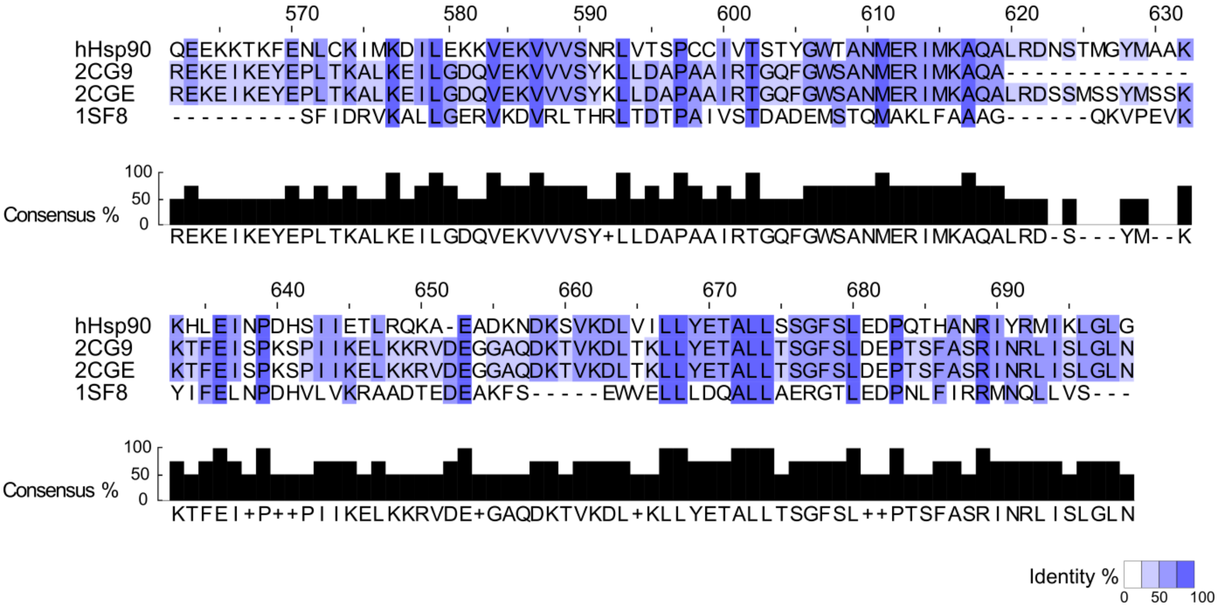

Figure S1: Multiple sequence alignment of Hsp90 CTD from *S. cerevisiae* (2CG9 and 2CGE), *E. coli* (1SF8), and *H. sapiens*. The sequence identity is represented with color on the sequences, ranging from blue (100%) to white (0%). The histograms located below the alignment show the overall consensus between the four sequences.

Validation of the homology model

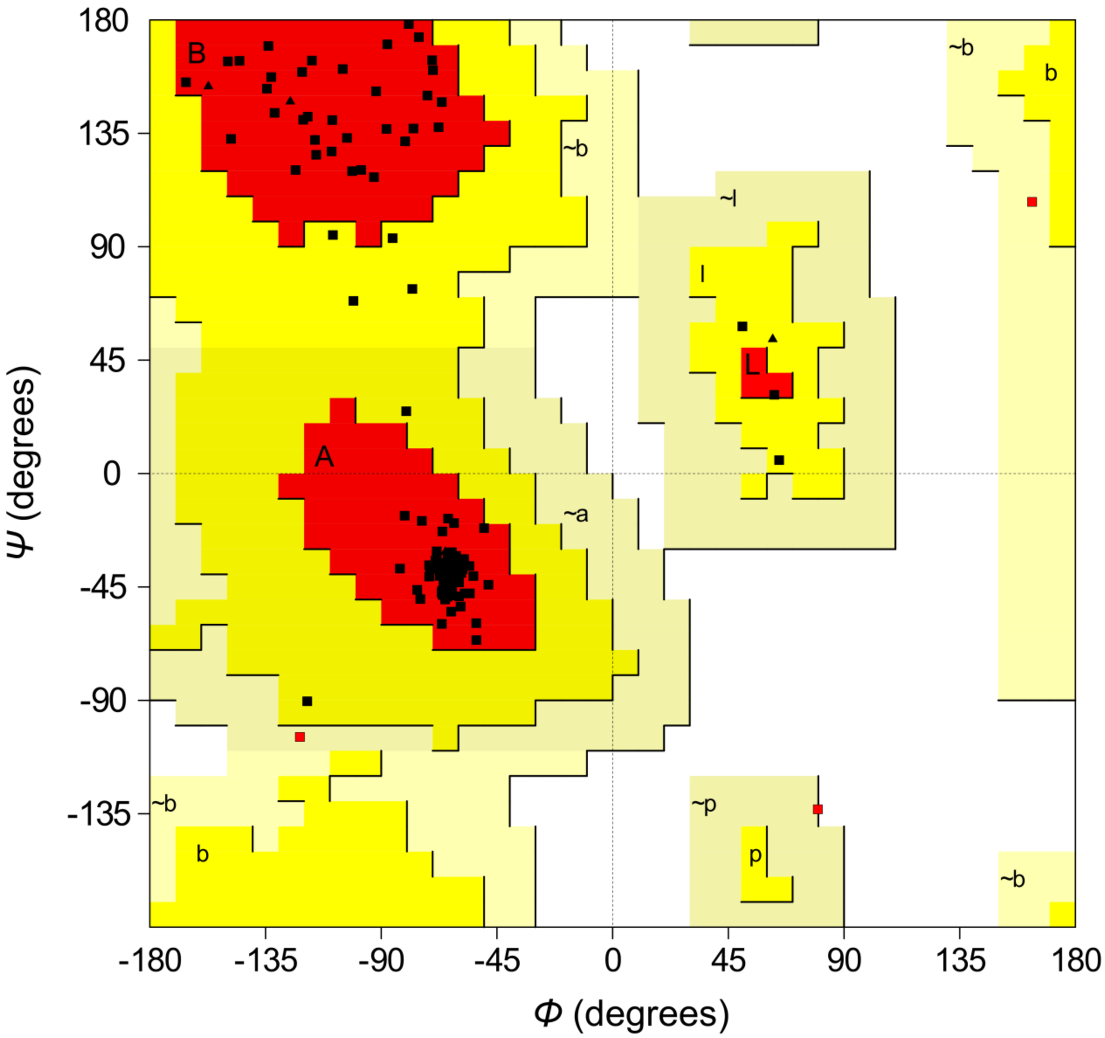

Figure S2: Ramachandran plot showing the ϕ/ψ torsion angles for all residues of the homology model. 91.4% of the residues are located in the most favorable regions of the plot (A, B, L), 6.2% of the residues are located in additionally allowed regions (a, b, l, p), and 2.3% in generously allowed regions (~a, ~ b, ~ l, ~ p) [3].

Structural deviations during MD simulations

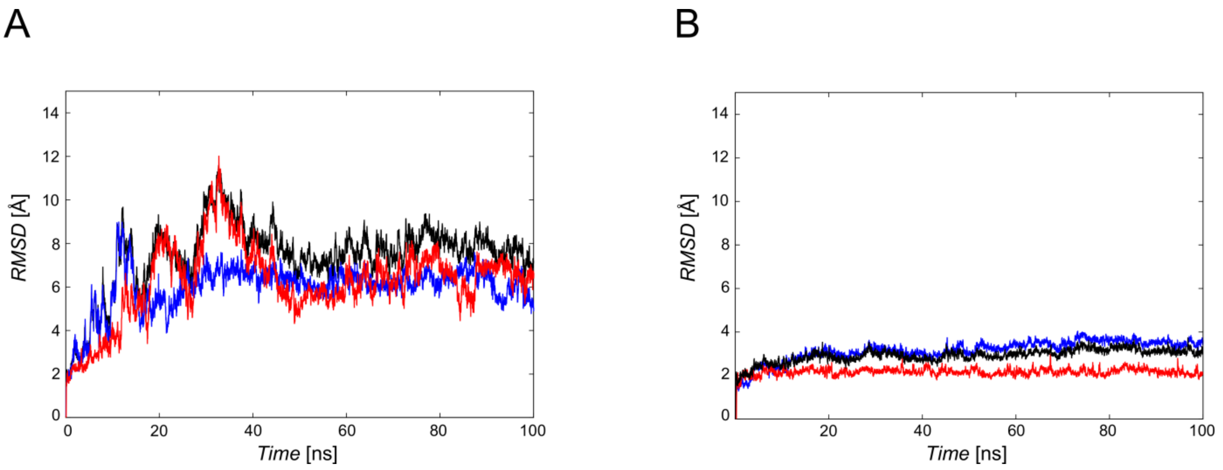

Figure S3: Root mean square deviations (RMSD) of backbone atoms during MD simulations of 100 ns length of hHSP90 CTD. (A) RMSD of the dimer (black) and single domains (Chain A, blue; Chain B, red). (B) RMSD of backbone atoms of dimer, chain A, and chain B (black, blue, red, respectively) calculated excluding helices H2 and H2‘.

Predicted hot spots for a CTD dimer of the crystal structure of an M-CTD construct of hHsp90

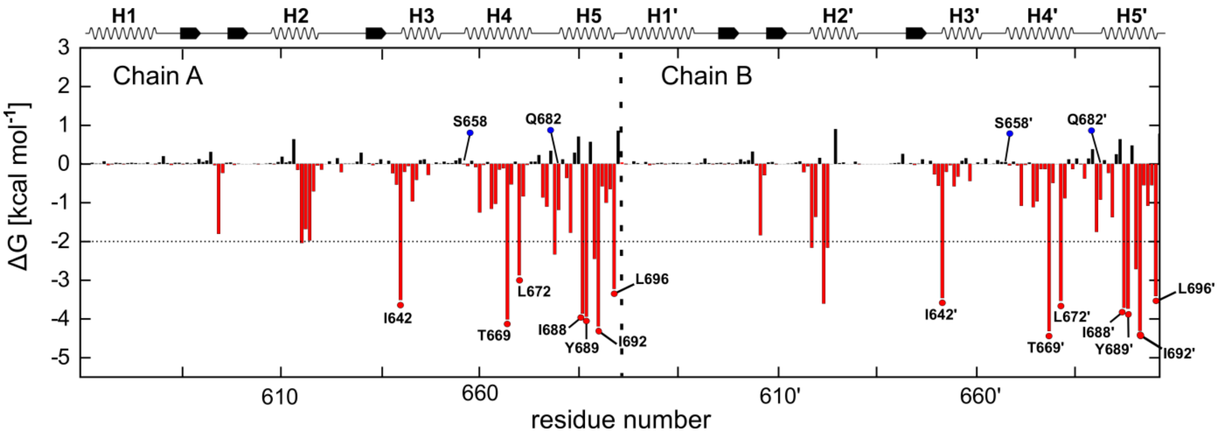

Figure S4: Contribution to the dimer stabilization of each amino acid within the hHsp90 CTD crystal structure (3Q6M). ΔG values are calculated by the MM-GB/SA approach [1,4] starting from the CTD dimer of the crystal structure of an M-CTD construct of hHsp90 (PDB code: 3Q6M) [5] and employing a structural decomposition of the effective energy [6]. The standard error in the mean is < 0.1 kcal mol^{-1} in all cases. Amino acids contributing to the dimerization with $\Delta G < -2$ kcal mol^{-1} are considered hot spots and are indicated in the graphic by red dots. The “cold spots” are marked with blue dots.

Hot spot prediction based on buried surface area

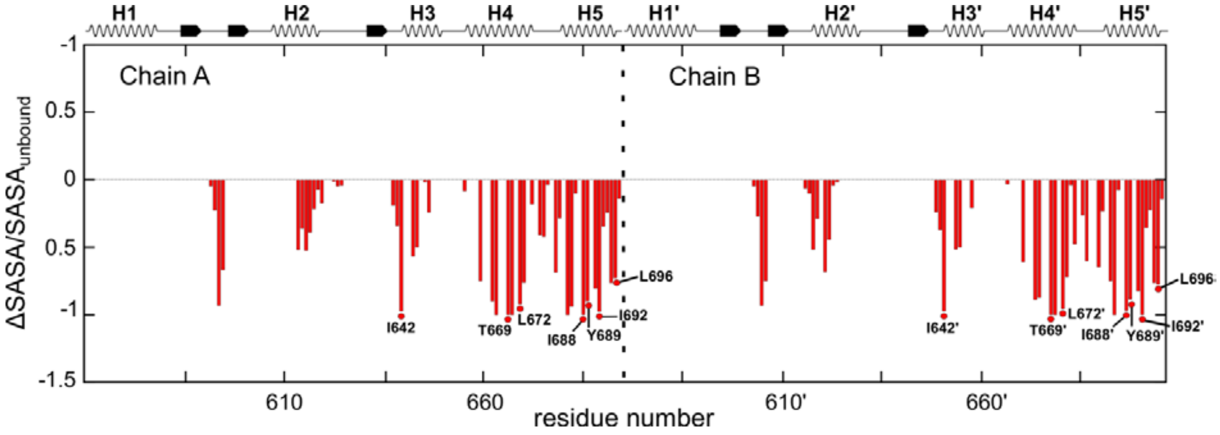

Figure S5: Residue-wise relative change in the buried surface area upon formation of the hHsp90 CTD dimer. For the calculations, the surface area values of the MM-GB/SA calculations starting from the CTD dimer of the crystal structure were used.

Thermofluor analysis of single alanine mutants of hHsp90 CTD

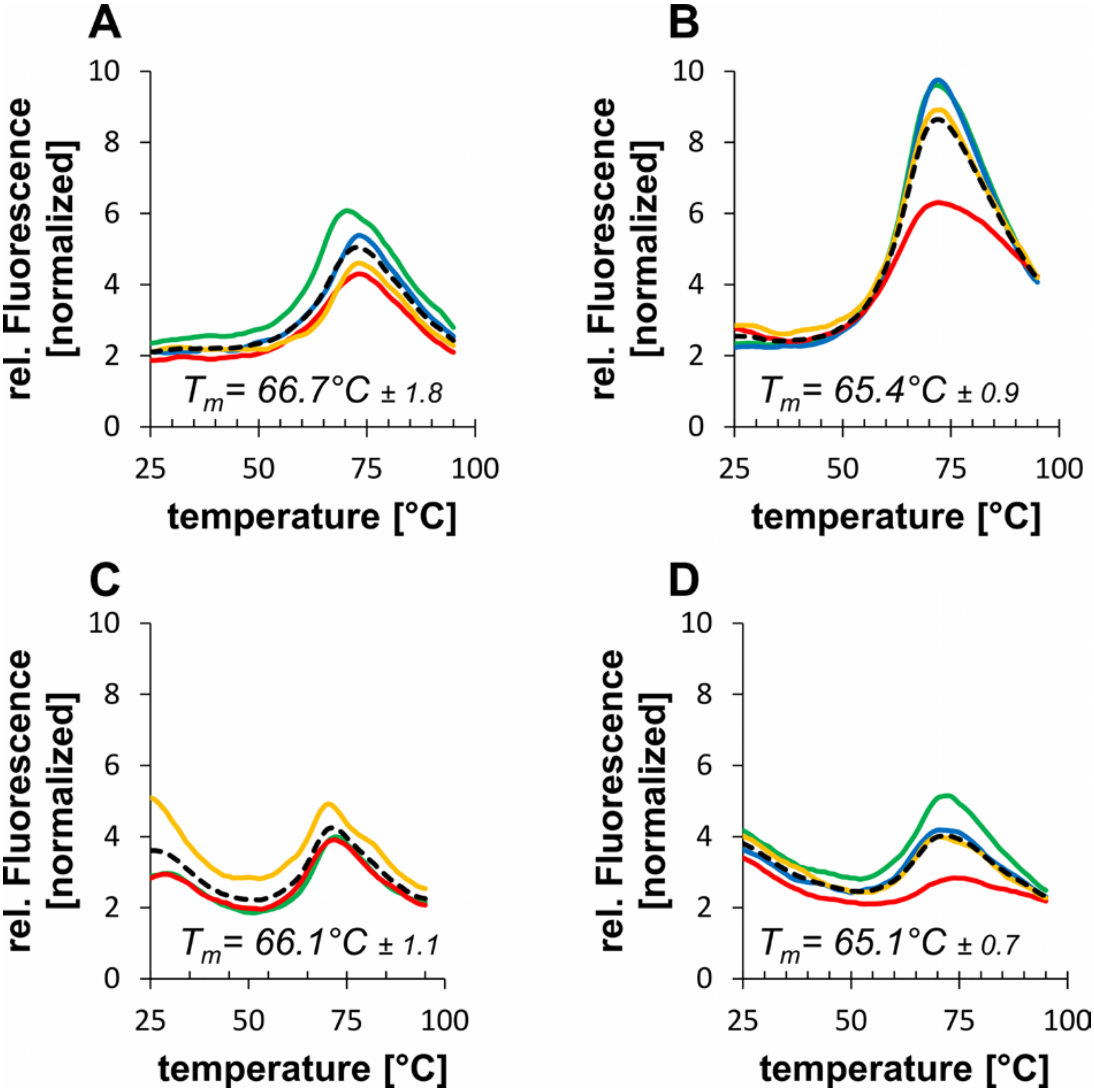

Figure S6: Thermofluor assay for investigating the stability of single alanine hHsp90 CTD mutants: Melting curves of measurements at pH 7.5 with the average T_m ± standard deviation are shown below the curves for the alanine single mutants I688A (A), Y689A (B), I692A (C), and L696A (D). The mean value (dotted black line) was calculated from three to four independent measurements (yellow, red, blue, green lines) in reaction buffer with 100 mM Tris.

CD spectroscopy measurements

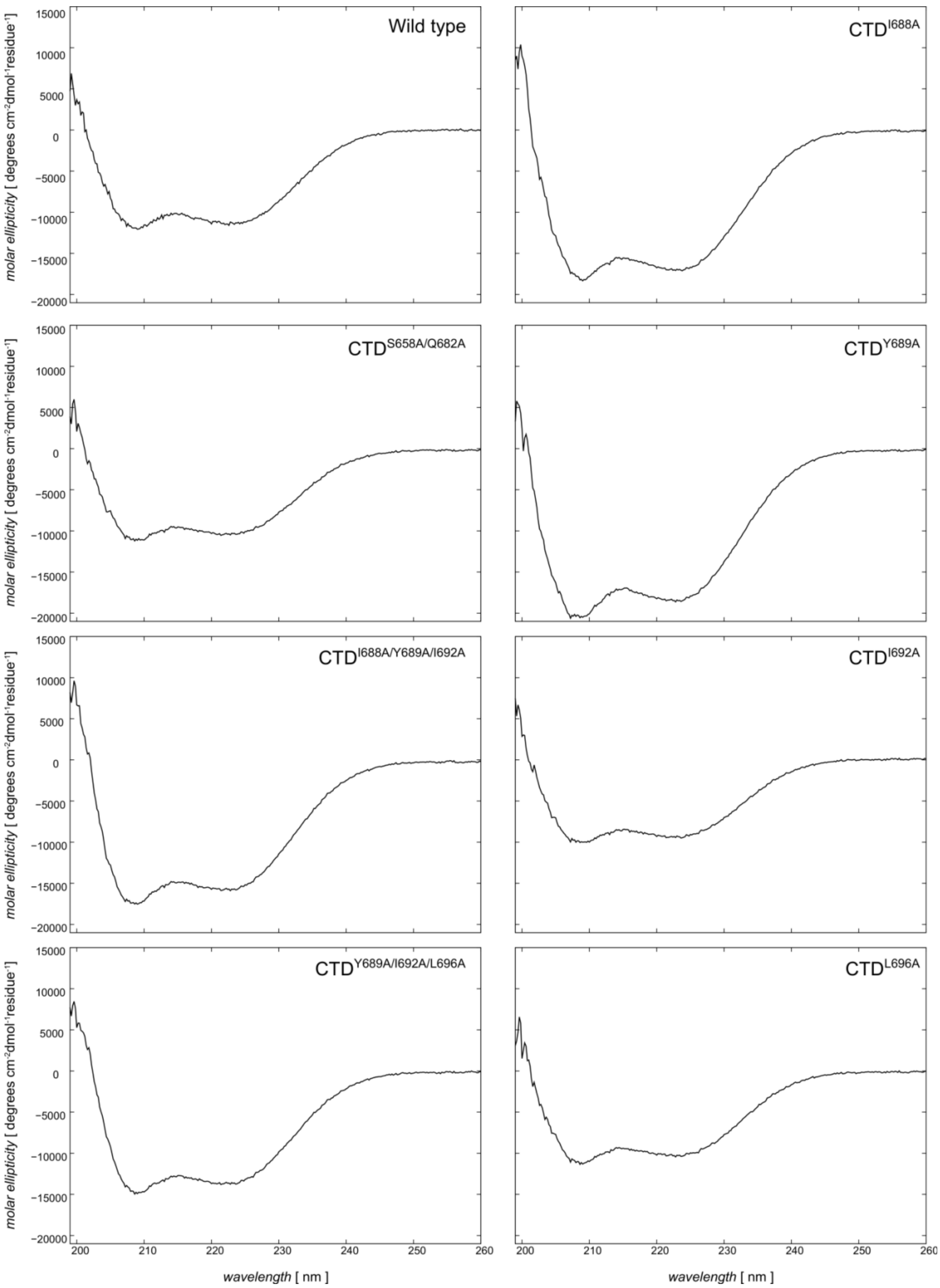

Figure S7: CD spectra of the hHsp90 CTD wild type and hHsp90 CTD mutants in the range 198-260 nm. The two pronounced peaks at about 207 and 225 nm reveal in all the cases the existence of a well-defined and mostly α-helical secondary structure.

Thermofluor analysis of hHsp90 CTD with ligands

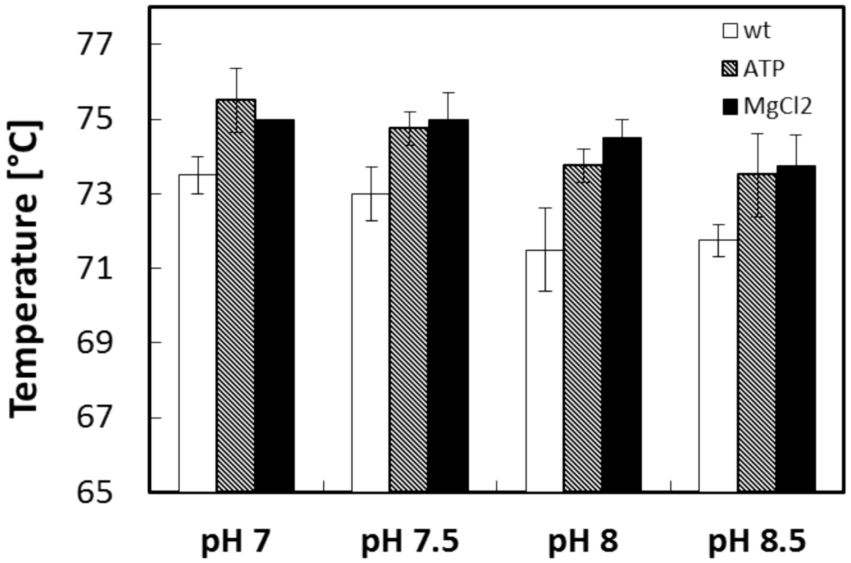

Figure S8: ATP and $MgCl_2$ effect on wild type hHsp90 CTD: Addition of 5 mM ATP (hatched) or 10 mM $MgCl_2$ (black) compared to the CTD of hHsp90 wild type (white). The mean value and standard deviation were calculated from four independent measurements in reaction buffer with 100 mM Tris at pH 7, 7.5 8 and 8.5 in °C.

Purification of CTD of hHsp90 variants

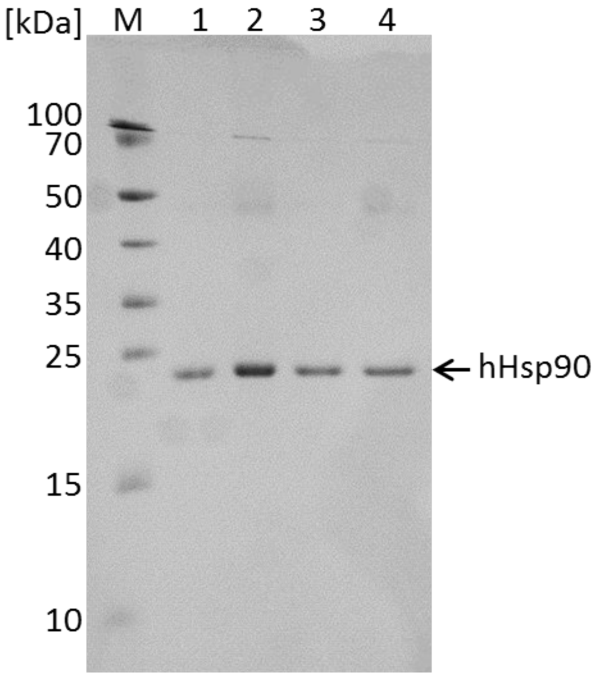

Figure S9: SDS-PAGE of Ni^{2+}-NTA purified CTD of hHsp90 variants: After expression and purification 1000 ng of wild type (1), $CTD^{Y689A/I692A/L696A}$ (2), $CTD^{I688A/Y689A/I692A}$ (3), and $CTD^{S658A/Q682A}$ (4) were solved in 5x LAP buffer [7]. Protein variants were analyzed on a 18% polyacrylamide gel with 2 µL of a protein standard (PageRuler™ Prestaind Protein Ladder; Thermo Scientific) and stained with colloidal Coomassie Blue [8]. Appearing protein bands (arrow) correspond to the molecular weight of 21.5 kDa (Table 1) for the CTD of hHsp90 variants indicating a pure protein solution.

Supplemental References

1. Massova I, Kollman PA (2000) Combined molecular mechanical and continuum solvent approach (MM-PBSA/GBSA) to predict ligand binding. Perspect Drug Discovery Des 18: 113-135.
2. Krüger DM, Gohlke H (2010) DrugScore(PPI) webserver: fast and accurate in silico alanine scanning for scoring protein-protein interactions. Nucleic Acids Res 38: W480-W486.
3. Laskowski RA, Macarthur MW, Moss DS, Thornton JM (1993) Procheck - a Program to Check the Stereochemical Quality of Protein Structures. J Appl Crystallogr 26: 283-291.
4. Homeyer N, Gohlke H (2012) Free Energy Calculations by the Molecular Mechanics Poisson-Boltzmann Surface Area Method. Molecular Informatics 31: 114-122.
5. Lee CC, Lin TW, Ko TP, Wang AH (2011) The hexameric structures of human heat shock protein 90. PLoS One 6: e19961.
6. Gohlke H, Kiel C, Case DA (2003) Insights into protein-protein binding by binding free energy calculation and free energy decomposition for the Ras-Raf and Ras-RalGDS complexes. J Mol Biol 330: 891-913.
7. Laemmli UK (1970) Cleavage of structural proteins during the assembly of the head of bacteriophage T4. Nature 227: 680-685.
8. Kang DH, Gho YS, Suh MK, Kang CH (2002) Highly sensitive and fast protein detection with coomassie brilliant blue in sodium dodecyl sulfate-polyacrylamide gel electrophoresis. Bull Korean Chem Soc 23: 1511-1512.
